# Supplementary material for: Consistency analysis of consciousness index and bispectral index in monitoring the depth of sevoflurane anesthesia in laparoscopic surgery
Source: PeerJ. 2024 Feb 14;12:e16848. doi: 10.7717/peerj.16848 (PMC10874172; doi:10.7717/peerj.16848)
Supplement: Supplemental Information 2 [file peerj-12-16848-s002.docx]

STROBE Statement—checklist of items that should be included in reports of observational studies

|  | Item No. | Recommendation | Page  No. | Relevant text from manuscript |
| --- | --- | --- | --- | --- |
| **Title and abstract** | 1 | (*a*) Indicate the study’s design with a commonly used term in the title or the abstract | 1 | To investigate the monitoring value of the Consciousness Index (IoC) for the depth of sevoflurane anesthesia in laparoscopic surgery. |
|  |  | (*b*) Provide in the abstract an informative and balanced summary of what was done and what was found | 1-2 | We conducted a retrospective study in Shaanxi Provincial People 's Hospital to analyze the effect of consciousness index on monitoring the depth of sevoflurane anesthesia and the consistency and accuracy of bispectral index monitoring sevoflurane anesthesia. |
| Introduction | | | |  |
| Background/rationale | 2 | Explain the scientific background and rationale for the investigation being reported | 2-3 | Bispectral index ( BIS ) is recognized as a sensitive and accurate objective index to evaluate the state of consciousness at home and abroad at this stage. The consciousness index is a new monitoring index of anesthesia depth reflecting the state of consciousness in our country. The research on monitoring the depth of anesthesia mainly focuses on propofol, but there are few reports on the effect of monitoring the depth of sevoflurane anesthesia and the consistency and accuracy of BIS and sevoflurane in maintaining anesthesia. |
| Objectives | 3 | State specific objectives, including any prespecified hypotheses |  | This study mainly analyzed the consistency of consciousness index and bispectral index during anesthesia induction, anesthesia maintenance to consciousness recovery. Receiver operating characteristic ( ROC ) curve was used to analyze the value of consciousness index and bispectral index in predicting the loss of consciousness during anesthesia induction and the recovery of consciousness during anesthesia recovery. |
| Methods | | | |  |
| Study design | 4 | Present key elements of study design early in the paper | 3 | In clinical practice, we found that the consciousness index is effective in monitoring the depth of anesthesia in laparoscopic surgery. |
| Setting | 5 | Describe the setting, locations, and relevant dates, including periods of recruitment, exposure, follow-up, and data collection | 3 | We conducted a retrospective study in Shaanxi Provincial People 's Hospital from April 2020 to June 2023. The clinical data of 108 patients undergoing laparoscopic hysterectomy or cholecystectomy under general anesthesia were collected. |
| Participants | 6 | (*a*) *Cohort study*—Give the eligibility criteria, and the sources and methods of selection of participants. Describe methods of follow-up  *Case-control study*—Give the eligibility criteria, and the sources and methods of case ascertainment and control selection. Give the rationale for the choice of cases and controls  *Cross-sectional study*—Give the eligibility criteria, and the sources and methods of selection of participants | 3 | The inclusion criteria of this study were : inclusion criteria : age > 18 years old ; laparoscopic hysterectomy or cholecystectomy under elective general anesthesia ; american Society of Anesthesiologists ( ASA ) grade I-II ; the expected operation time was less than 6 hours. The surgical data were complete. Exclusion criteria : those who are allergic to anesthetic drugs ; patients with severe heart, lung and kidney dysfunction ; complicated with severe cardiovascular and cerebrovascular diseases ; mental disorders ; long-term use of sedative drugs or sleeping drugs. |
|  |  | (*b*) *Cohort study*—For matched studies, give matching criteria and number of exposed and unexposed  *Case-control study*—For matched studies, give matching criteria and the number of controls per case |  |  |
| Variables | 7 | Clearly define all outcomes, exposures, predictors, potential confounders, and effect modifiers. Give diagnostic criteria, if applicable | 4-5 | A total of 108 patients undergoing elective laparoscopic hysterectomy or cholecystectomy under general anesthesia in our hospital from April 2020 to June 2023 were selected. At the same time, the domestic anesthesia depth monitor and the bispectral index monitor were used to monitor the consciousness index ( IoC ) and the bispectral index ( BIS ) respectively. The BIS and IoC at different time points of the operation were recorded, and the consistency between IoC and BIS was analyzed. |
| Data sources/ measurement | 8* | For each variable of interest, give sources of data and details of methods of assessment (measurement). Describe comparability of assessment methods if there is more than one group |  | N/A |
| Bias | 9 | Describe any efforts to address potential sources of bias |  | N/A |
| Study size | 10 | Explain how the study size was arrived at |  | The clinical data of 108 patients undergoing elective laparoscopic hysterectomy or cholecystectomy under general anesthesia in our hospital from April 2020 to June 2023 were collected through electronic medical records. |

Continued on next page

| Quantitative variables | 11 | Explain how quantitative variables were handled in the analyses. If applicable, describe which groupings were chosen and why |  | N/A |
| --- | --- | --- | --- | --- |
| Statistical methods | 12 | (*a*) Describe all statistical methods, including those used to control for confounding | 6 | SPSS22.0 software was used for data analysis. The measurement data were expressed as ( mean ± standard deviation ), and the t test was used for comparison. The count data were expressed as ' cases ' or ' % ', and the chi-square test was used. If the theoretical frequency was ≤ 5 but ≥ 1, the chi-square value needed to be corrected. If the theoretical frequency was < 1, Fisher 's exact test was used. Bland-Altman consistency test was used to analyze the consistency of the two anesthesia depth indexes, and the mean difference and 95 % limit of agreement ( LOA ) of the two indexes were calculated. ROC curve was used to analyze the predictive value of BIS and IoC on patients ' consciousness. P < 0.05 indicated that the difference was statistically significant. |
|  |  | (*b*) Describe any methods used to examine subgroups and interactions |  |  |
|  |  | (*c*) Explain how missing data were addressed |  |  |
|  |  | (*d*) *Cohort study*—If applicable, explain how loss to follow-up was addressed  *Case-control study*—If applicable, explain how matching of cases and controls was addressed  *Cross-sectional study*—If applicable, describe analytical methods taking account of sampling strategy |  |  |
|  |  | (*e*) Describe any sensitivity analyses |  |  |
| Results | | | | |
| Participants | 13* | (a) Report numbers of individuals at each stage of study—eg numbers potentially eligible, examined for eligibility, confirmed eligible, included in the study, completing follow-up, and analysed | 6 | Through electronic medical records, the clinical data of 108 patients undergoing elective laparoscopic hysterectomy or cholecystectomy under general anesthesia were finally collected. |
|  |  | (b) Give reasons for non-participation at each stage | 6 | 108 patients met the inclusion criteria of this study. |
|  |  | (c) Consider use of a flow diagram |  |  |
| Descriptive data | 14* | (a) Give characteristics of study participants (eg demographic, clinical, social) and information on exposures and potential confounders |  |  |
|  |  | (b) Indicate number of participants with missing data for each variable of interest |  |  |
|  |  | (c) *Cohort study*—Summarise follow-up time (eg, average and total amount) |  |  |
| Outcome data | 15* | *Cohort study*—Report numbers of outcome events or summary measures over time |  |  |
|  |  | *Case-control study—*Report numbers in each exposure category, or summary measures of exposure |  |  |
|  |  | *Cross-sectional study—*Report numbers of outcome events or summary measures |  |  |
| Main results | 16 | (*a*) Give unadjusted estimates and, if applicable, confounder-adjusted estimates and their precision (eg, 95% confidence interval). Make clear which confounders were adjusted for and why they were included | 6-7 | The mean value of the difference between BIS and IoC was 1.3 ( 95 % LOA : -53.4 ~ 56.0 ) from the beginning of anesthesia induction to the end of propofol anesthesia induction. There was no significant difference between the two methods ( P = 0.628 ), suggesting that IoC and BIS had good consistency during anesthesia induction. |
|  |  | (*b*) Report category boundaries when continuous variables were categorized |  |  |
|  |  | (*c*) If relevant, consider translating estimates of relative risk into absolute risk for a meaningful time period |  |  |

Continued on next page

| Other analyses | 17 | Report other analyses done—eg analyses of subgroups and interactions, and sensitivity analyses |  | N/A |
| --- | --- | --- | --- | --- |
| Discussion | | | | |
| Key results | 18 | Summarise key results with reference to study objectives | 7-8 | The mean value of the difference between BIS and IoC was 1.3 from before anesthesia induction to the completion of propofol anesthesia induction. From sevoflurane inhalation to consciousness recovery, the average difference between BIS and IoC was 0.3.There was no significant difference between the two measurement methods during anesthesia induction and anesthesia maintenance to consciousness recovery ( P > 0.05 ). ROC curve analysis showed that the area under the curve ( AUC ) of BIS and IoC monitoring loss of consciousness was 0.967 and 0.959 respectively, and the optimal cut-off values were 81 and 77, respectively. The AUC of BIS and IoC were 0.995and 0.963, respectively. |
| Limitations | 19 | Discuss limitations of the study, taking into account sources of potential bias or imprecision. Discuss both direction and magnitude of any potential bias | 9 | There are some shortcomings in this study, such as no monitoring of muscle relaxation, muscle relaxation on the depth of anesthesia monitoring also has an impact ; at the same time, the type of surgery selected is laparoscopic cholecystectomy or uterine adnexectomy, which does not represent the entire laparoscopic surgery, resulting in the promotion and application of IoC monitoring anesthesia depth is limited. |
| Interpretation | 20 | Give a cautious overall interpretation of results considering objectives, limitations, multiplicity of analyses, results from similar studies, and other relevant evidence | 9 | The depth of anesthesia monitored by IoC during the maintenance of sevoflurane anesthesia is comparable to BIS, and it is in good agreement with BIS intraoperative monitoring of the depth of anesthesia during the maintenance of sevoflurane anesthesia. |
| Generalisability | 21 | Discuss the generalisability (external validity) of the study results | 9 | This study also observed the BIS and IoC values at different time points. The results showed that the IoC value was significantly lower than the BIS value at T2 ~ T9, and the difference was statistically significant, which may be related to the different sensitivity of BIS and IoC to opioids. |
| Other information | |  | | |
| Funding | 22 | Give the source of funding and the role of the funders for the present study and, if applicable, for the original study on which the present article is based |  | N/A |

*Give information separately for cases and controls in case-control studies and, if applicable, for exposed and unexposed groups in cohort and cross-sectional studies.

**Note:** An Explanation and Elaboration article discusses each checklist item and gives methodological background and published examples of transparent reporting. The STROBE checklist is best used in conjunction with this article (freely available on the Web sites of PLoS Medicine at http://www.plosmedicine.org/, Annals of Internal Medicine at http://www.annals.org/, and Epidemiology at http://www.epidem.com/). Information on the STROBE Initiative is available at www.strobe-statement.org.
